# Chance-level hit rates in closed-set, forced-choice audiometry and a novel utility for the significance test-based detection of malingering

**DOI:** 10.1371/journal.pone.0231715

**Published:** 2020-04-21

**Authors:** Thomas Steffens, Lisa M. Steffens, Steven C. Marcrum

**Affiliations:** 1 Department of Otolaryngology, University Hospital Regensburg, Regensburg, Germany; 2 Center for Cognitive Sciences, University of Bremen, Bremen, Germany; Medical University Hannover; Cluster of Excellence Hearing4all, GERMANY

## Abstract

The primary aim of this study was to extend existing theory on the relationship between chance-level performance and the number of alternatives and trials in closed-set, forced-choice speech audiometry and sound localization methods. When calculating chance performance for closed-set, forced-choice experiments with multiple trials, the binomial distribution should be preferred over the simple 1/*a* probability, as the latter is appropriate only for single trial experiments. The historical use of constant hit rates for determining chance performance has been based upon the assumption that random hits are distributed evenly across multiple trials. For any closed-set, forced-choice task with 2 to 10 alternatives and 2 to 100 trials, we calculated the probability of obtaining any given hit rate due to random guessing alone according to the binomial distribution. Hit rates with probabilities p > 0.05 were interpreted as being likely to occur due to random chance alone, whereas hit rates with probabilities of p ≤ 0.05 were interpreted as being unlikely to occur due to chance alone. For sound localization experiments with speakers at fixed positions, the expected probability of a random hit was also calculated using the binomial distribution. The expected angular root mean square (rms) error in sound localization resulting from the random selection of sound sources was investigated using Monte Carlo simulations. A new aspect in the interpretation of test results was identified for situations in which the observed number of hits is much lower than would be expected due to chance alone. For test methods incorporating a relatively low number of alternatives and a sufficiently high, yet clinically feasible, number of trials, both upper and lower thresholds for chance-level performance could be identified. This lower threshold represents the lowest hit rate which can be expected through random chance alone. Extending interpretation of results to include this lower threshold affords the ability to not only identify performance significantly superior to that of chance, but also that significantly poorer than chance and thereby represents a simple method for the objective detection of malingering.

## Introduction

Audiometry involves the use of listening tasks to assess a person’s ability to detect, discriminate, localize or recognize various aspects of acoustic stimuli. Though comprising some of the most ecologically valid methods within the standard audiological test battery, both speech audiometry and sound localization tasks are nonetheless affected by a number of factors independent of the listener’s hearing status. One factor, which despite its relevance has received relatively limited attention, is the likelihood that the listener might correctly perform a given task by chance alone. This test characteristic is especially influential when implementing closed-set, forced-choice methodologies, such as is routinely done in both clinical and research capacities for patients of all ages. In contrast to open-set speech audiometry, where the listener is tasked with producing one response out of effectively unlimited alternatives, in closed-set, forced-choice speech testing, possible responses are limited to a small number of known response options. As the number of response options (alternatives) is limited and the listener is required to provide a response for every trial (forced-choice), the probability of randomly guessing the correct response (hit) may become significant.

As the number of alternatives falls, the probability of correctly performing a task by chance alone rises. A test result can be interpreted as being superior to chance performance if the proportion of hits (hit rate) is higher than the chance level. Traditionally, chance level has been defined as a fixed probability, calculated from the reciprocal of the number of alternatives (1/*a*). However, the nature of this relationship is more dynamic than is commonly assumed. Only in instances of a single trial or infinitely many trials does chance-level performance equal the reciprocal of the number of alternatives. This is due to variation in the distribution of random hits across multiple trials. Rather than being constant, the number of hits achieved by guessing will change from multiple trial assessment to multiple trial assessment, varying around the chance-level probability of a single trial (1/*a*). It is of some concern, then, that in the overwhelming majority of publications using closed-set, forced-choice methods with multiple trials, the chance level hit rates are misinterpreted to be constant and identical with the probability of success in a single trial (1/*a*), irrespective of the number of trails conducted in the test. Examples of well-cited papers accepting this assumption are [1–3], to name a few. Additionally, it has even been used to define the lower asymptote of closed-set speech intelligibility functions [4].

The primary aim of this study was to extend existing theory on the relationship between chance-level performance and the number of alternatives and trials in closed-set, forced-choice speech audiometry and sound localization methods through recognition of the random nature of guessing across multiple trials. This extension is grounded in the mathematics of the binomial distribution, wherein its standard deviation describes the inherent scatter of hit rates achieved by chance in multiple trial measurements, which occur with certain probabilities based of the binomial distribution. Applying the binomial distribution, the influence of chance on the test results can be calculated to get an exact probability of chance occurrence for each test result. It is applicable to all closed-set, forced-choice assessments with binary outcomes (right or wrong, true or false, 1 or 0) and multiple trials.

Leveraging properties of the binominal distribution to interpret speech audiometry results is not entirely new. Hagerman was the first to use the binomial distribution in modeling the reliability of speech audiometry [5] and Thornton and Raffin, producing likely the most-cited paper on the topic, described the calculation of significant critical differences of two speech audiometry results based on the confidence limits of binomial distributions [6]. Carney and Schlauch revised the results of Thornton and Raffin, which were based on the theoretical framework of the binomial distribution, by applying additional computer simulations of random error using the binomial distribution [7]. The simulation of possible test results improved the precision of the calculation of critical differences, because such calculations can also be used in cases in which the statistical properties of some measures are not fully known. For example, Thornton and Raffin used an approximation to the variance of difference scores to construct their table. Gelfand proposed a method to optimize the reliability of speech audiometric scores, also on the basis of the binomial distribution, by increasing the number of items by counting every correct syllable of a word individually instead of the whole word [8]. The method presented in this paper is different than those previous methods because it is designed to assist in the improved estimation of chance performance and objective detection of malingering in closed-set tasks, whereas the previous works have been aimed towards identifying thresholds for significant differences between repeated speech recognition assessments. Our method can therefore be applied for diverse audiometric purposes, such as interpreting results of closed-set, n-alternative forced-choice word recognition testing or localization experiments with a limited number of loudspeakers. Additionally, the methods presented in this study provide the clinician with a means not only to identify performance significantly superior to that of chance, but also to identify performance significantly poorer than chance, a clinically-meaningful capability given that some patients might feel incentivized to deliberately provide incorrect responses (malingering). The new method presented here also helps to identify unintentional, systematic errors on the part of listeners as well as technical problems through the objective basis of statistical significance testing, and extends the portfolio of methods in clinical decision-making.

The following sections provide a brief introduction to the theory and application of the binomial distribution in closed-set audiometric procedures. Generally, the probability of randomly selecting a correct answer (hit) from a given set of alternative response options in a single trial has been defined as *p* = *1/a*, where *a* is the number of available response options. As such, the probability of hits by random guessing remains constant for all single trials within a multiple-trial, closed-set, forced-choice test, as long as *a* does not change and unless there is a systematic influence of effects such as learning or fatigue across assessments (which in this work we assume will not occur). The probability distribution of such a binary random variable which takes the value 1 (hit, success) with probability *p* and the value 0 (miss, wrong answer) with probability *q = 1 − p* is subject to the Bernoulli distribution, which is a special case of the binomial distribution for a single trial (*n = 1*, Bernoulli trial).

For multiple independent Bernoulli trials (*1 < n < ∞*), the binomial distribution results in probabilities of hits due to chance which depend on the number of alternatives (*a*) and the number of trials (*n*). Even if the probability of a hit by chance is constant in every single trial and the inherent probability of hits due to chance does not vary, the measured number or proportion of hits (hit rate) due to chance certainly varies across many multiple-trial assessments. This variation is a fundamental property of chance. Hence, in multiple-trial assessments, the number of hits or the hit rate by chance should not be assumed to be constant, calculated according to the probability of success in a single trial (*1/a*). This holds only for a single trial or an infinite number of trials (The Law of Large numbers for Bernoulli Trials), but not for small data sets with a limited number of trials. Rather, in multiple-trial measures, the single-trial probability *1/a* is the mean (expected) hit rate by chance (see Eq 6). But the hit rates between multiple multi-trial assessments with the same numbers of alternatives and trials vary randomly around this mean. The result is an interval of possible hit rates by chance with different probabilities around the mean hit rate, corresponding to the standard deviation of the binomial distribution. A significant advantage of recognizing the random nature of random guessing using the binomial model arises from the possibility of calculating exact probabilities for various chance-based hit rates depending on the number of trials and alternatives. Hence, the probability of observing a given hit rate due to chance alone can be calculated and the question of whether a certain test result is significantly influenced by chance can be answered with the well-known methods of single-sided or two-sided interpretations of significance.

To calculate the probability of observing a given hit rate due to chance alone as a binomial variable, the hit rate *H* will be defined according to the textbooks of statistic as the proportion *k* of hits relative to the number of trials *n* (e.g. number of presented single-word stimuli)

$$H=\frac{k}{n}$$ (1)

and the probability *p* of success due to chance for a single trial as

$$p=\frac{1}{a}.$$ (2)

The probability *P(k)* of observing a hit rate *H* of *k* hits in *n* trials purely by chance is given by the probability mass function of the binomial distribution

P(k)=(nk)pk(1−p)n−k, (3)

for *k = 0*, *1*, *2*, *…*, *n*, with

(nk)=n!k!(n−k)!. (4)

The mean number of hits (k_*μ*_) of the binomial distribution is

$$k_{\mu }=n∙p$$ (5)

and the mean hit rate *H*_*μ*_ is equal to the single trial success probability *p*:

$$H_{\mu }=\frac{k_{\mu }}{n}=\frac{n∙p}{n}=p.$$ (6)

Using Eqs 2 and 3, the exact probability *P(k)* for *k* observed hits by chance of assessments with different numbers of alternatives *a* and trials *n* can be calculated.

With the calculated probability of the random occurrence of a given hit rate, both one-sided and two-sided tests of significance can be performed to identify results with low-likelihood of having occurred due to chance alone. In this study, for any combination of *a* and *n*, in one-sided testing the largest hit rate by chance with *p* > 0.05 is defined as the upper threshold of random guessing *(U)*. Usefully, for certain combinations of *a* and *n*, an additional lower threshold *(L)* for hit rates by chance, for which *p* > 0.05, can also be identified. In the case of two-sided testing, the critical value of *p* will be *p* > 0.025, because the probability of *p* > 0.05 is divided into the upper and lower tail of the binomial distribution. In single-sided testing, only observed hit rates outside one side of the interval between upper or lower boundaries are of interest, which occur with chance probabilities of p ≤ 0.05, so that they deviate significantly from the expected hit rates on the basis of purely random guessing. In two-sided testing, observed hit rates outside either the upper or lower boundaries are relevant and *p* ≤ 0.05 is the sum of the probabilities for significantly deviating hit rates outside the interval divided into p ≤ 0.025 each for hit rates above the upper or below the lower boundary.

In this way, two clinically relevant observations are made possible by single-sided testing of significance: First, if the observed hit rate is above *U*, the result can be interpreted as being significantly better than chance. Second, if the proportion of observed hits is lower than *L*, the result will be significantly lower than chance and malingering or systematic errors on the part of the equipment, the examiner, or the listener, might be suspected. Sometimes, a listener is not willing to perform correctly in a closed-set, forced-choice task and deliberately selects. This behavior can be detected by the fact that, with a sufficiently high number of trials and a small number of alternatives, a certain number of hits can be expected by chance with a probability > 0.05. In this way, the lower threshold *L* meaningfully increases the interpretative power of closed-set methods because malingering or aggravation of hearing loss can be detected with the precision of statistical significance.

In the first part of this paper, *U* and *L* are calculated for chance-level hit rates for typically used numbers of alternatives and trials in closed-set, forced-choice audiometric methods and for root mean square (RMS) errors in closed-set, forced-choice sound-source localization experiments. In the second part, the proposed method of significance testing for malingering will be presented.

## Methods

For any closed-set, forced-choice task with 2 to 10 alternatives and 2 to 100 trials, we calculate the probability of hit rates due to random guessing using Eq 3. The absolute number of hits (*k*) is transformed into hit rates *H* by Eq 1.

The decision as to whether observed hit rates were significantly free of chance can be based either on single-sided (one-tailed) or two-sided (two-tailed) tests of significance. Hit rates with chance probabilities *P(k)* > 0.05 will be interpreted as significantly influenced by chance in single-sided testing. Hence, hit rates with chance probabilities of *P(k)* ≤ 0.05 will be interpreted as significantly free of chance. For certain combinations of number of alternatives (*a*) and number of trials (*n*), the lower threshold (*L*) of random guessing is the smallest hit rate *H* for which the chance probability is *P(k)* > 0.05. The upper threshold (*U*) is defined as the largest hit rate with *P(k)* > 0.05. Single-sided testing is used when the observed results may differ from those expected by chance in only one direction. For example, single-sided significance testing would be appropriate if one were only interested in cases with hit rates either larger or lower than *U*. This is the case in the vast majority of clinical issues. In this example, the probability for randomly guessed hit rates either higher than *U* or lower than *L* would be *P(k)* ≤ 0.05 in each case.

Two-sided testing can be conducted when results in both possible directions are of interest. That is to say, when one is interested in both hit rates larger than *U* or smaller than *L*. For two-sided statistics, the total probability of randomly guessed hit rates both above *U* and below *L* is *P(k)* ≤ 0.05, which yields *P(k)* ≤ 0.025 for each threshold. The higher and lower thresholds, *U* and *L*, are therefore the largest and smallest hit rates which can be achieved by chance alone with a probability of *P(k)* > 0.025 for each of the thresholds. Observed hit rates higher than *U* or lower than *L*, thereby occurring with a probability of *P(k)* ≤ 0.05 for each threshold in the case of single-sided testing and *P(k)* ≤ 0.025 in the case of two-sided testing, will be defined as being significantly independent of chance.

*U* and *L* will also be calculated for RMS errors by pure chance in sound-source localization experiments applying Monte Carlo simulations. In the first step, one speaker will be set as the target sound source out of the number *a* of available speakers. Then a random selection is made from the available loudspeakers, which is simulated by the binomial distribution (Eq 3) with the probability *p = 1/a* of a random hit. If random selection by the binomial distribution corresponds to a hit in the current simulation run, the next run is started until a predefined number of failed attempts are performed. If the random selection is incorrect (no hit), an incorrectly selected loudspeaker is determined by a random number distribution with uniform probability of *1/a* in a second selection run (response). Now it has to be checked that the set and the randomly selected loudspeaker (response) are actually different, otherwise a new random selection will be made. Only in case of a wrong random selection, the angle between the set and the wrong selected speaker will be determined as angle error. This loop is repeated until the given number of *n* trials with false responses has been reached. In the end, the simulated random angle errors will be averaged as RMS error over the number of trials. Different Monte Carlo simulations were carried out for typical combinations of numbers of speakers, angles between them and numbers of trials. For each configuration of speakers and number of trials, the number of simulated subjects was 100,000 to calculate a reasonable exact forecast of random RMS errors. *U* and *L* were calculated from the parameter of the resulting distribution of RMS errors to get the upper and lower thresholds of RMS errors for single- and two-sided tests of significance. The Monte Carlo simulation was performed with PTC Mathcad Prime 5.0 (PTC, Boston MA).

## Results

### Upper (U) and lower (L) thresholds of hit rates by chance

An example calculation of single-sided and two-sided probabilities of hit rates by pure random guessing for a test with four alternatives and 20 trials is given in Table 1.

**Table 1:** Probabilities P(k) of k-hits and the resulting hit rates by pure random guessing for a test with four alternatives and 20 trials. Hit rates with P(k) ≤ 0.05 (dark grey fields) are interpreted as significant different from random guessing in single-sided and P(k) ≤ 0.025 (light grey fields) in two-sided tests of significance.

a = 4 alternatives, n = 20 trials		
k-Hits	Hit Rate	P(k)
**0**	**0,00**	0,003**
**1**	**0,05**	0,021**
2	0,10	0,067
3	0,15	0,134
4	0,20	0,190
5	0,25	0,202
6	0,30	0,169
7	0,35	0,112
8	0,40	0,061
**9**	**0,45**	0,027*
**10**	**0,50**	0,010**
**11**	**0,55**	0,003**
**12**	**0,60**	0,001**
**13**	**0,65**	0,000**
**14**	**0,70**	0,000**
**15**	**0,75**	0,000**
**16**	**0,80**	0,000**
**17**	**0,85**	0,000**
**18**	**0,90**	0,000**
**19**	**0,95**	0,000**
**20**	**1,00**	0,000**

**Fig 1:**
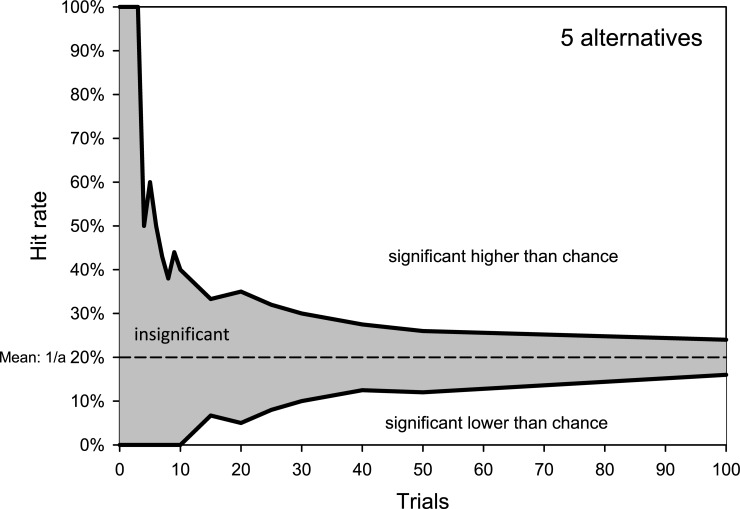
Upper and lower thresholds (black lines) of hit rates by chance (p > 0.05, single-sided testing) for closed-sets with 5 alternatives. Only observed hit rates higher than the upper or lower than the lower thresholds are significantly free of guessing (p ≤ 0.05). The mean of the distribution of hit rates by chance which is used by the traditional 1/a-rule gives a constant probability of random guessing of p = 0,2 (broken line) and is at the middle of the insignificant results area. The higher the number of trials the narrower the insignificant results area.

The complete set of *U* and *L* of hit rates by chance for tests with 2 to 10 alternatives and 2 to 100 trials for single-sided testing is presented in Table 2. If an observed hit rate for a certain combination of number of trials *n* (rows) and alternatives *a* (columns) is either higher than *U* or lower than *L*, the result can objectively be interpreted as being significantly different from results which would be expected due to chance alone. The probability of observing hit rates either higher than U or lower than *L* by chance alone is P(k) ≤ 0.05. *U* and *L* for two-sided testing are presented in Table 3. Two-sided assessments of results are of significant clinical interest, as hit rates both lower than *L* and higher than *U* can be considered valid and meaningful. For example, a given participant’s hit rate can be lower than, higher than, or between the thresholds for random guessing. In those measurements, the sum of the probabilities for hit rates by chance less than *L* and greater than *U* is P(k) ≤ 0.05. Hence, the overall probability of P(k) ≤ 0.05 for hit rates outside the expected range for chance results is divided between the upper and lower thresholds, resulting in P(k) ≤ 0.025 for any threshold. Two-sided testing increases the range of hit rates possible through chance alone (e.g. Table 3), as compared with single-sided testing (e.g. Table 2). In general, the higher the number of trials, the smaller the difference between the upper and lower thresholds and, consequently, the range of hit rates possible by chance. Fig 1 displays example results of the decreasing range of hit rates by chance for increasing numbers of trials (2–100) for a test setup consisting of five alternative responses. The narrowing range of insignificant results demonstrates the trend towards the mean *(1/a)* of the probability distribution for increasing numbers of trials. At the same time, *L* increases from zero and opens a range for results below *L* that deviate significantly from the hit rates by chance.

**Table 2:** *One-sided testing*: Lower (L) and upper (U) thresholds (limits) of the distribution of insignificant different hit rates from random guessing (p > 0.05) for *one-sided testing* and different numbers of alternatives (a) and number of trials (n). To reach results significantly free of random guessing, the observed hit rates must be higher than U or lower than L.

	a = 2		a = 3		a = 4		a = 5		a = 6		a = 7		a = 8		a = 9		a = 10	
**n**	L	U	L	U	L	U	L	U	L	U	L	U	L	U	L	U	L	U
**2**	0.000	1.000	0.000	1.000	0.000	1.000	0.000	0.500	0.000	0.500	0.000	0.500	0.000	0.500	0.000	0.500	0.000	0.500
**4**	0.000	1.000	0.000	0.750	0.000	0.500	0.000	0.500	0.000	0.500	0.000	0.500	0.000	0.500	0.000	0.500	0.000	0.250
**6**	0.167	0.833	0.000	0.667	0.000	0.500	0.000	0.500	0.000	0.500	0.000	0.333	0.000	0.333	0.000	0.333	0.000	0.333
**8**	0.250	0.750	0.125	0.625	0.000	0.500	0.000	0.375	0.000	0.375	0.000	0.375	0.000	0.375	0.000	0.250	0.000	0.250
**10**	0.300	0.700	0.100	0.600	0.000	0.500	0.000	0.400	0.000	0.400	0.000	0.300	0.000	0.300	0.000	0.300	0.000	0.300
**15**	0.333	0.667	0.133	0.533	0.067	0.400	0.067	0.333	0.000	0.333	0.000	0.267	0.000	0.267	0.000	0.267	0.000	0.200
**20**	0.350	0.650	0.200	0.500	0.100	0.400	0.050	0.350	0.050	0.300	0.050	0.250	0.000	0.250	0.000	0.200	0.000	0.200
**25**	0.360	0.640	0.200	0.440	0.120	0.360	0.080	0.320	0.040	0.280	0.040	0.240	0.040	0.240	0.000	0.200	0.000	0.200
**30**	0.367	0.633	0.233	0.433	0.133	0.367	0.100	0.300	0.067	0.267	0.067	0.233	0.033	0.200	0.033	0.200	0.033	0.167
**40**	0.400	0.600	0.250	0.425	0.150	0.325	0.125	0.275	0.080	0.250	0.075	0.225	0.050	0.200	0.050	0.180	0.025	0.175
**50**	0.420	0.580	0.260	0.400	0.180	0.320	0.120	0.260	0.100	0.210	0.080	0.200	0.060	0.180	0.040	0.180	0.040	0.160
**100**	0.460	0.540	0.290	0.380	0.210	0.290	0.160	0.240	0.120	0.210	0.100	0.180	0.080	0.160	0.070	0.150	0.060	0.140

**Table 3:** *Two-sided testing*: Lower (L) and upper (U) thresholds (limits) of the distribution of insignificant different hit rates (p > 0.05) for *two-sided testing* and different numbers of alternatives (a) and number of trials (n). To reach results significantly free of random guessing, the observed hit rates must be higher than U or lower than L.

	**a = 2**	**a = 3**		**a = 4**		**a = 5**		**a = 6**		**a = 7**		**a = 8**		**a = 9**		**a = 10**	
n	L	U	L	U	L	U	L	U	L	U	L	U	L	U	L	U	L	U
**2**	0.000	1.000	0.000	1.000	0.000	1.000	0.000	1.000	0.000	1.000	0.000	0.500	0.000	0.500	0.000	0.500	0.000	0.500
**4**	0.000	1.000	0.000	0.750	0.000	0.750	0.000	0.750	0.000	0.500	0.000	0.500	0.000	0.500	0.000	0.500	0.000	0.500
**6**	0.167	0.833	0.000	0.833	0.000	0.667	0.000	0.500	0.000	0.500	0.000	0.500	0.000	0.500	0.000	0.333	0.000	0.333
**8**	0.125	0.875	0.000	0.630	0.000	0.500	0.000	0.500	0.000	0.500	0.000	0.375	0.000	0.375	0.000	0.375	0.000	0.375
**10**	0.200	0.800	0.100	0.600	0.000	0.500	0.000	0.500	0.000	0.400	0.000	0.400	0.000	0.300	0.000	0.300	0.000	0.300
**15**	0.267	0.733	0.133	0.533	0.067	0.467	0.000	0.400	0.000	0.333	0.000	0.333	0.000	0.267	0.000	0.267	0.000	0.267
**20**	0.300	0.700	0.150	0.500	0.100	0.450	0.050	0.350	0.000	0.350	0.000	0.300	0.000	0.250	0.000	0.250	0.000	0.250
**25**	0.320	0.680	0.160	0.480	0.120	0.400	0.080	0.360	0.040	0.320	0.040	0.280	0.000	0.240	0.000	0.200	0.000	0.200
**30**	0.333	0.667	0.200	0.467	0.100	0.400	0.067	0.333	0.067	0.300	0.033	0.267	0.033	0.233	0.000	0.200	0.000	0.200
**40**	0.375	0.625	0.200	0.450	0.125	0.375	0.100	0.300	0.080	0.275	0.050	0.250	0.050	0.225	0.025	0.200	0.025	0.200
**50**	0.380	0.630	0.220	0.440	0.140	0.360	0.100	0.300	0.080	0.260	0.060	0.220	0.040	0.200	0.040	0.200	0.002	0.180
**100**	0.430	0.570	0.260	0.400	0.190	0.310	0.140	0.260	0.110	0.220	0.090	0.200	0.070	0.180	0.060	0.160	0.050	0.150

**Fig 2:**
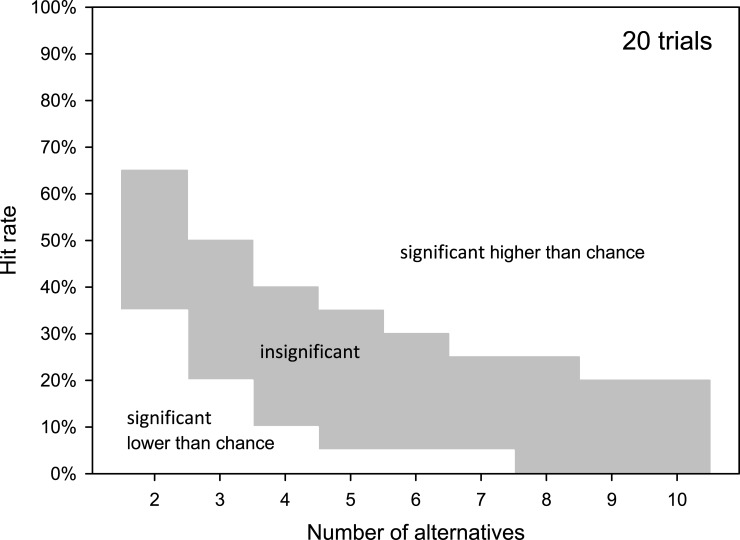
Dependency of results area for hit rates with a probability of random guessing of p > 0.05 (gray area) on the number of alternatives for 20 trials and single-sided testing. For low numbers of alternatives (2 to 7) a lower threshold of insignificant results exists (lower limit of gray area). For 8 and more alternatives a hit rate of 0% is also possible by pure random guessing with a probability of p > 0.05. Results within the white areas can be interpreted as significantly free from random guessing (p ≤ 0.05).

**Fig 3:**
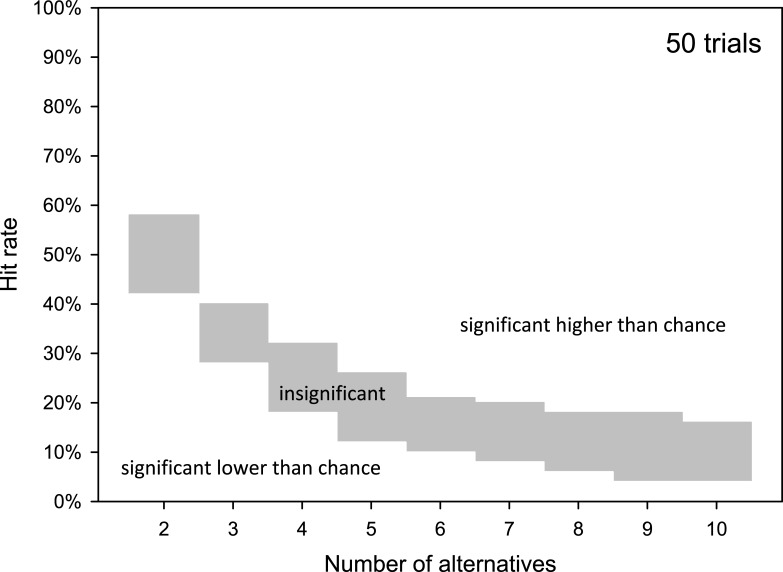
Dependency of results area for hit rates with a probability of random guessing of p > 0.05 (gray area) on the number of alternatives for 50 trials and single-sided testing.

**Fig 4:**
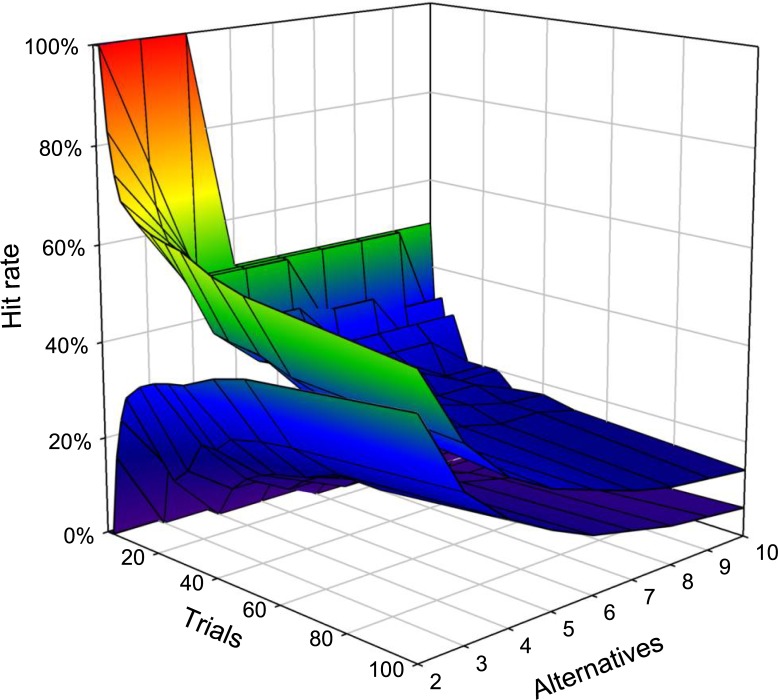
Course of U and L as a function of the number of alternatives and trials.

Lower thresholds (*L*), which represent the dividing lines separating results high enough to be potentially due to chance from those so poor that they are likely independent of chance, only occurred in cases where the number of alternative responses was low and the number of trials was high. Fig 2 demonstrates the general relationship between the number of alternative responses (*a*) and the *U* and *L*. For a closed-set speech audiometry assessment consisting of 20 words (20 independent trials) and 2–10 alternative responses possible per trial, the probability of no hits (hit rate = 0) due to chance was P(k) ≤ 0.05 in single-sided testing when 2–7 alternatives were provided. As the number of alternatives decreased, the probability of random hits increased and results with no or very few chance-related hits became less common. Even in the absence of audibility, a listener is required by the nature of forced-choice testing to select a response. If this selection can be assumed to occur randomly, the listener should successfully complete the task at *p* > 0.05. A complete lack of hits is to be expected only in instances of technical malfunction, misunderstanding of the task, or intentionally poor performance on the part of the listener. The range of significant results below the lower threshold can be increased for the same number of alternatives by increasing the number of trials. Fig 3 gives an example for a test utilizing 50 trials. The lower range of significant results is markedly extended and the range of insignificant results is decreased in comparison to 20 trials (see Fig 2). The connected course of the relationship between the two variables, the number of alternatives and trials, is shown in Fig 4 as a 3D plot of U and L.

### Application for the interpretation of localization testing

The potential clinical benefit of incorporating the hit rates’ true scatter with the binomial model presented in this study, as opposed to the commonly used constant hit rate by chance model (1/*a*), was evaluated by a re-evaluation of published data describing the localization abilities of children with bilateral cochlear implants [9]. The primary task in that study consisted of n = 30 trials in which the sequentially-implanted children were asked to identify a target loudspeaker out of a = 3 alternatives. The children were evaluated in 3 listening conditions: 1) binaural, or using both cochlear implants, 2) monaural, using only the first-implanted ear, and 3) monaural, using only the second-implanted ear.

**Fig 5:**
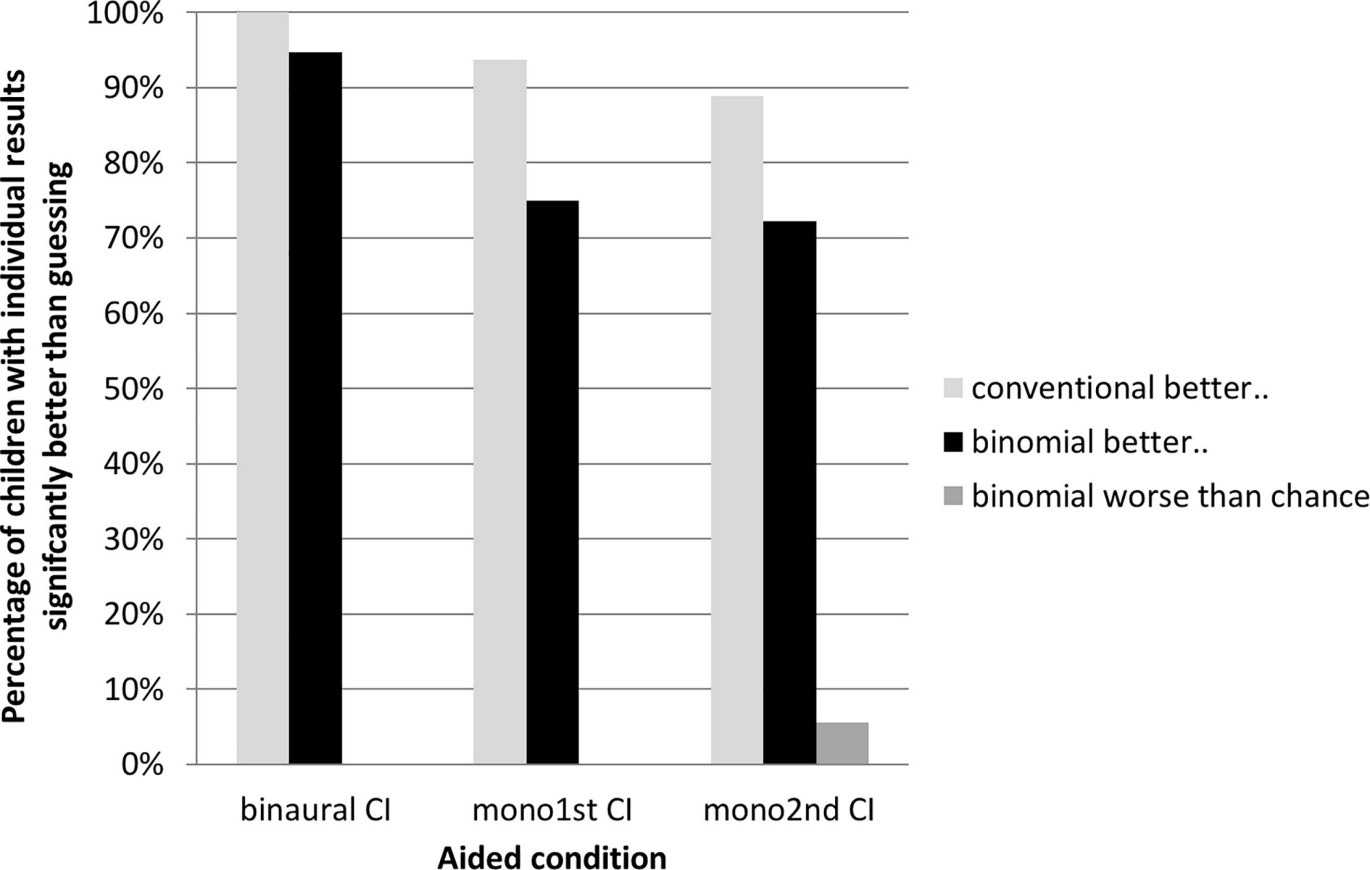
Proportion of children with results significantly better than chance in a localization test in sequentially cochlear implanted children (reevaluation of data from [9]). Three alternative loud speakers (90° separated) had to be identified in 30 trials for each of three aided conditions (binaural CI: with both implanted ears, mono1st CI: unilateral with early implanted ear, mono2nd CI: unilateral with late implanted ear). Criteria of individual results significantly better than chance where due to the conventional 1/3 probability of chance (light gray bars) and the more precise binomial model (black and dark grey bars). A remarkably lower proportion of children had significant better scores than expected by chance calculated with the binomial model in comparison to the conventional 1/3 probability. In case of unilateral localization with the later implanted second CI (mono2nd CI), a significant lower proportion of children performed better than chance according to the binomial model. One child performed significant worse with the late implanted second implant alone, maybe because of unwillingness or fatigue.

In the re-evaluation, the proportion of test results significantly better or worse than would be expected by chance alone was determined for each condition (Fig 5). The criteria for significance were defined either by the binomial model with *a* = 3 and *n* = 30 or the conventional (1/*a*) model. The binomial limits for significantly better results (single-sided testing) were found to be 0.433 for *U* and 0.233 for *L*. Therefore, only results outside these limits can be considered significantly independent of chance. The conventional model resulted in a single, constant threshold of 0.33, with results above this threshold being described as significantly better than chance.

The proportion of children with results which could be described as significantly better than chance was lower for the binomial model than the conventional model for all 3 listening conditions, a difference which suggests that clinical interpretation of the dataset depends in large part on the manner in which random chance is modelled. For example, of the 18 children who completed testing when using the most recently implanted cochlear implant monaurally (mono 2nd CI), 16 performed better than the conventional chance threshold of 0.33. More precise results obtained using the binomial model and single-sided testing reveal performance significantly better than chance in only 13 ears. Therefore, the individual probability of a child to significantly improve localization with a second implant was 16/18 (89%) in the instance of constant chance level modelling and 13/18 (72%) using the more precise binomial model. It is possible then, that use of the conventional model resulted in an overestimation of the localization abilities and the probability of individual improvements of the children in this test condition. The finding that the most commonly used calculation method overestimates the number of better than chance results in comparison to the interval of hit rates by chance of the binomial distribution can be generalized.

### RMS angular error in localization experiments

As a result of a localization measurement, not only the hit rates of the correctly detected loudspeakers can be evaluated, but also the RMS (root mean square) angle errors. Here, the angle between the target and the incorrectly selected loudspeaker is determined and the RMS error is calculated over several trials. In the Monte Carlo simulation, the angle error expected by random guessing is calculated with the hit probability from the binomial distribution. If the test runs are repeated several times in multi-trial experiments, this results in an interval of angle RMS errors with defined probabilities. From this, *L* and *U* for random RMS errors were calculated for popular loudspeaker arrangements and one-sided and two-sided testing (Table 4). These limits can be used to determine whether observed RMS errors are significantly different from the values expected for random guessing.

**Table 4:** Lower (L) and upper (U) thresholds of insignificant RMS errors for single- and two-sided testing as a function of the number of loudspeakers and trials. The total angle span of the speaker arrangement was 180° or 140° (angle span) and the speakers are separated by angles between 90° and 10° (angle between speakers). Every speaker is to be set as target and the random selection is repeated 2 to 20 times (repetitions). The number of trials (test runs) is determined by the product of the number of loudspeakers and the number of repetitions.

number of speakers	angel span	angel between speakers	repetitions	trials	mean RMS error	stand. dev.	single-sided testing		two-sided testing	
							L	U	L	U
**3**	180°	90°	2	**6**	101,0°	23,9°	**61,7°**	**140,4°**	**54,2°**	**147,9°**
**3**	180°	90°	3	**9**	102,0°	19,1°	**70,7°**	**133,5°**	**64,7°**	**139,6°**
**3**	180°	90°	5	**15**	103,0°	14,6°	**79,0°**	**126,9°**	**74,4°**	**131,5°**
**3**	180°	90°	10	**30**	103,5°	10,2°	**86,7°**	**120,3°**	**83,5°**	**123,5°**
**3**	180°	90°	20	**60**	103,7°	7,1°	**91,9°**	**115,4°**	**89,7°**	**117,7°**
**5**	180°	45°	2	**10**	88,6°	15,9°	**62,5°**	**114,7°**	**57,5°**	**119,7°**
**5**	180°	45°	3	**15**	89,1°	12,8°	**68,0°**	**110,2°**	**63,9°**	**114,2°**
**5**	180°	45°	5	**25**	89,4°	9,9°	**73,2°**	**105,6°**	**70,1°**	**108,8°**
**5**	180°	45°	10	**50**	89,7°	6,9°	**78,3°**	**101,2°**	**76,1°**	**103,4°**
**5**	180°	45°	20	**100**	89,8°	4,9°	**81,8°**	**97,9°**	**80,2°**	**81,8°**
**19**	180°	10°	2	**38**	80,6°	10,1°	**64,0°**	**97,2°**	**60,8°**	**100,4°**
**19**	180°	10°	3	**57**	80,8°	8,2°	**67,4°**	**94,3°**	**64,8°**	**96,8°**
**19**	180°	10°	5	**95**	81,0°	6,3°	**70,6°**	**91,4°**	**68,7°**	**93,4°**
**19**	180°	10°	10	**190**	81,1°	4,4°	**73,8°**	**88,4°**	**72,4°**	**89,8°**
**19**	180°	10°	20	**380**	81,2°	3,1°	**76,0°**	**86,3°**	**75,0°**	**87,3°**
**15**	140°	10°	2	**30**	60,8°	6,1°	**50,7°**	**70,9°**	**48,7°**	**72,8°**
**15**	140°	10°	3	**45**	60,9°	5,0°	**52,6°**	**69,1°**	**51,1°**	**70,7°**
**15**	140°	10°	5	**75**	61,0°	3,9°	**54,6°**	**67,4°**	**53,4°**	**68,6°**
**15**	140°	10°	10	**150**	61,0°	2,7°	**56,5°**	**65,6°**	**55,6°**	**66,4°**
**15**	140°	10°	20	**300**	61,1°	1,9°	**57,9°**	**64,2°**	**57,3°**	**64,8°**

## Discussion

Randomly selecting 1 of *a* alternative targets in any given trial is subject to the Bernoulli distribution. For measurements with multiple trials (*n >* 1), however, the Bernoulli distribution changes into the binomial distribution and the observed hit rates guided by chance alone vary randomly from assessment to assessment. The conventional use of the constant hit rate 1/*a*, independent of the number of trials, is only accurate for instances in which either only 1 or infinitely many trials are conducted. In the latter case, the binomial probability converges to 1/*a*. In assessments with a limited number of trials, such as is common to clinical audiometric testing, the random hit rate is not constant, but rather varies according to the standard deviation of the binomial distribution. An advantage of recognizing the influence of the binomial distribution is that it provides an exact probability for observed hit rates in cases of pure random guessing. Thus, significance testing can be performed to determine whether a result is objectively better or worse than would be expected by chance alone. In the majority of closed-set, forced-choice audiometry studies with multiple trials, all hit rates above the single trial chance level of 1/*a* have been erroneously considered significantly free of random effects and thus valid. In this study, however, recognition of the variable effectiveness of random guessing allowed for the definition of thresholds (U and L), which allow for a precise objective, test-specific distinction between results significantly affected by chance from those significantly different from the results expected by chance alone.

### A new significance test-based method for the objective detection of malingering

The investigation and interpretation of hit rates better than chance probability is common practice. However, it is often neglected to interpret hit rates that are lower than those that can be expected by chance. Application of the binomial distribution allows for a new significance test-based method for the objective interpretation of results below chance levels. With the binomial model, it is possible that a carefully balanced combination of number of trials and alternatives provides two thresholds for hit rates, *U* and *L*, beyond which the results deviate significantly from hit rates by pure guessing. The upper threshold *U* determines the maximum hit rate. However, the lower threshold L, which determines the minimum hit rate to be expected solely from random guessing, is of particular importance. This is a new aspect in the interpretation of chance-level performance that leads to more information through the application of the binomial distribution. For a clinical trial, it would therefore be advisable to use test conditions where both U and L can be used to interpret the test results. Doing so offers a clinically useful advantage, as the lower threshold can be applied as a means of assessing results for malingering or systematic technical or procedural errors. For instances in which closed-set speech audiometry is used for the purpose of determining the extent of hearing damage for a work-related injury case, for example, this method provides an objective means for the clinician to better interpret both good and poor results. Obtaining results significantly poorer than would be expected due to chance alone (below L) might be an indication that a patient is simulating a complete loss of speech understanding by intentionally selecting incorrect answers. The method would also be beneficial in differentiating between patients with poor speech understanding abilities and those performing poorly due to lack of understanding of the task. Irrespective of the cause of the poor performance, for closed-set, forced-choice tests with the optimized characteristics of low alternative and high trial numbers, the lower threshold of guessing (L) represents a valuable, new source of information for the statistically valid interpretation of speech audiometry results.

The following example illustrates the benefit of the lower threshold for significant different hit rates from random guessing. During the localization tests, one child stood out due to severe fatigue. He had to be especially motivated for the last test (monaural listening with 2nd CI). His test result was then so low that it was well below the lower limit of the hit rate by chance, L (Fig 5). This gives a strong indication that this test result should not be considered in the evaluation and can be identified and treated as an outlier generated by individual factors.

In addition to clinical use, utilization of the binomial model also influences interpretation of research results. Fig 5 demonstrates how the interpretation of test results can be meaningfully affected through use of the random hit rate interval between U and L. A smaller proportion of children was identified as having performed better than would be expected by chance alone when using the binomial model thresholds U and L, as compared to the constant 1/*a* model (13/18 vs. 16/18). This finding suggests that the effect of sequential cochlear implantation on lateralization performance might have been substantially overestimated if the unrealistic constant hit rate equal to the chance probability 1/*a* of a single trial is assumed for multi-trial measurements.

The use of constant hit rates to define chance-level performance in multi-trial closed-set, forced-choice testing is based upon the untenable assumption that randomness does not apply to hit rates across trials. By accounting for the random distribution of success and failure across trials, the clinician is provided with a simple, objective tool to further support decision-making.

## Conclusion

The conventional handling of random guessing in closed-set, forced-choice testing as a constant variable should be limited to single-trial methods. In conventionally multi-trial methods, like speech audiometry or localization experiments with many words or sound directions per assessment, the probability of hits by pure guessing varies randomly. This results in an range of possible hit rates by chance that can be calculated from the binomial distribution and which depend upon both the number of alternatives and the number of trials. The use of the binomial distribution adds more exact probabilities for hit rates or RMS localization errors by random guessing. Using certain combinations of low numbers of alternatives and sufficently high, yet clinically practical, numbers of trials leads to a lower threshold of hit rates or RMS localization errors than is likely to be achieved by guessing alone. Hit rates or RMS localization errors smaller than these lower limits can be interpreted as significantly free of random guessing. This may allow the simulation of a severe hearing disorder to be detected with the precision of tests of significance. In this way the application of the binomial model extends the interpretation of multi-trial, closed-set methods.
